# Engineering of Zinc Finger Nucleases Through Structural Modeling Improves Genome Editing Efficiency in Cells

**DOI:** 10.1002/advs.202310255

**Published:** 2024-04-10

**Authors:** Shota Katayama, Masahiro Watanabe, Yoshio Kato, Wataru Nomura, Takashi Yamamoto

**Affiliations:** ^1^ Genome Editing Innovation Center Hiroshima University Higashi‐Hiroshima 739‐0046 Japan; ^2^ Research Institute for Sustainable Chemistry National Institute of Advanced Industrial Science and Technology (AIST) Higashi‐Hiroshima 739‐0046 Japan; ^3^ Biomedical Research Institute National Institute of Advanced Industrial Science and Technology (AIST) Ibaraki 305‐8566 Japan; ^4^ Graduate School of Biomedical and Health Sciences Hiroshima University Hiroshima 734‐8553 Japan; ^5^ Division of Integrated Sciences for Life Graduate School of Integrated Sciences for Life Hiroshima University Higashi‐Hiroshima 739‐8526 Japan

**Keywords:** genome editing, protein engineering, structural biology, zinc finger nucleases

## Abstract

Genome Editing is widely used in biomedical research and medicine. Zinc finger nucleases (ZFNs) are smaller in size than transcription activator‐like effector (TALE) nucleases (TALENs) and CRISPR‐Cas9. Therefore, ZFN‐encoding DNAs can be easily packaged into a viral vector with limited cargo space, such as adeno‐associated virus (AAV) vectors, for in vivo and clinical applications. ZFNs have great potential for translational research and clinical use. However, constructing functional ZFNs and improving their genome editing efficiency is extremely difficult. Here, the efficient construction of functional ZFNs and the improvement of their genome editing efficiency using AlphaFold, Coot, and Rosetta are described. Plasmids encoding ZFNs consisting of six fingers using publicly available zinc‐finger resources are assembled. Two functional ZFNs from the ten ZFNs tested are successfully obtained. Furthermore, the engineering of ZFNs using AlphaFold, Coot, or Rosetta increases the efficiency of genome editing by 5%, demonstrating the effectiveness of engineering ZFNs based on structural modeling.

## Graphical abstract

Zinc finger nucleases (ZFNs) have great potential for translational research and clinical use. The efficient construction of functional ZFNs and the improvement of their genome editing efficiency using AlphaFold, Coot, and Rosetta are succeeded.

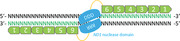

## 1 Introduction

Genome editing tools are broadly useful in research and medicine. Among these, transcription activator‐like effector (TALE) nucleases (TALENs) and CRISPR‐Cas9 can be easily constructed and used.^[^ ^1^ ^]^ However, TALENs and CRISPR‐Cas9 patents are active until 2032^[^ ^2^ ^]^ and 2033,^[^ ^3^ ^]^ respectively.^[^ ^4^ ^]^ In contrast, patents for zinc finger nucleases (ZFNs) already expired in 2020.^[^ ^5^ ^]^

An arrayed ZF (0.3–0.6 kbp) is smaller in size than an arrayed TALE (encoded in 1.7–2 kbp DNA) or Cas9 (4.1 kbp).^[^ ^6^ ^]^ Therefore, ZFN‐encoding DNAs can be easily packaged into an adeno‐associated virus (AAV) vector with a limited cargo space (≈4.6 kb).^[^ ^7^ ^]^ Instead of AAV/CRISPR‐Cas9,^[^ ^8^ ^]^ AAV/ZFN provides a more powerful platform for in vivo gene therapy. ZFNs have potential applications in in vivo gene therapy.

ZFNs consist of two functional units, a DNA‐binding moiety and a catalytic moiety. The assembled ZF protein functions as a DNA‐binding moiety, whereas the FokI nuclease functions as a catalytic moiety. However, at present, a FokI heterodimer nuclease (ELD/KKR),^[^ ^9^ ^]^ whose patent was active until 2031,^[^ ^10^ ^]^ is widely used. To avoid the industrial use of a FokI heterodimer nuclease (ELD/KKR), an ND1 heterodimer nuclease (DDD/RRR) was developed by our research group in 2019.^[^ ^11^ ^]^ In this study, we aimed to create ZF‐ND1 for genome editing in cells by linking an ND1 heterodimer nuclease (DDD/RRR) to assemble ZF proteins.

An assembled ZF protein consists of three tandem ZF modules,^[^ ^12^ ^]^ each of which recognizes three DNA bases. ZFNs function as heterodimeric nucleases; therefore, they recognize 18 DNA bases. Selection‐based methods can be used to construct assembled ZF proteins.^[^ ^13^ ^]^ However, these methods are labor‐intensive and time‐consuming. An alternative method for constructing assembled ZF proteins is the assembly of ZF modules using standard molecular biology techniques.^[^ ^14^ ^]^ This method provides researchers with a much easier method to construct assembled ZF proteins. However, modularly assembled ZFNs have a small number of functional ZFN pairs (≈94% failure rate for the ZFN pairs tested^[^ ^15^ ^]^).^[^ ^16^ ^]^ Sangamo Therapeutics has its own modular assembly systems for easily constructing functional ZF proteins consisting of five or six tandemly arrayed ZF modules.^[^ ^17^ ^]^ We hypothesized that the modular assembly of the ZF modules would be useful for constructing 5 or 6‐finger ZFNs.

In recent years, the accuracy of protein structures has been determined using the AI‐based protein structural modeling tool AlphaFold.^[^ ^18^ ^]^ To address the elucidation of 6‐finger ZF (6‐ZF) and DNA interactions, we modeled the 6‐ZF‐DNA structure using biomolecule modeling tools such as AlphaFold, Coot,^[^ ^19^ ^]^ and Rosetta.^[^ ^20^ ^]^ Rosetta has a specific application in protein‐DNA modeling named the RosettaDNA application.^[^ ^21^ ^]^ RosettaDNA can be used to design protein‐DNA interface structures to improve binding affinity through a residue‐directed design that arbitrarily modifies specific amino acid residues. In addition, the X‐ray crystal structure of the murine transcription factor Zif268 is well‐characterized as a C_2_H_2_‐type ZF, and its DNA complex has been determined.^[^ ^22^ ^]^ Therefore, we attempted to build and modify the complex model of 6‐ZF‐DNA based on the Zif268‐DNA structure.

In this study, we created 6‐finger ZFNs using a modular assembly of ZF modules and obtained functional ZFNs. Furthermore, we succeeded in improving genome‐editing efficiency through structural modeling using AlphaFold, Coot, and Rosetta.

## 2 Results

### 2.1 Development of Functional ZF‐ND1 Pairs

**Figure 1:**
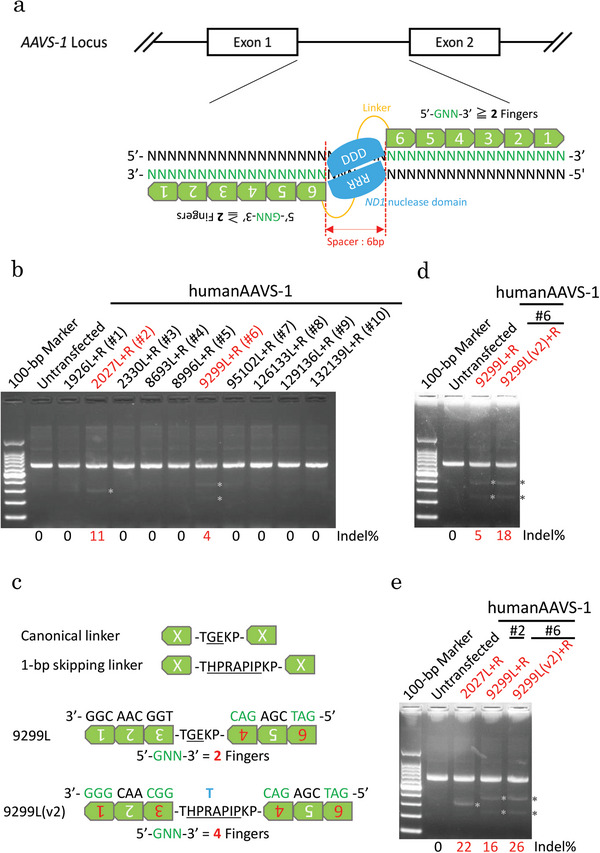
Development of ZF‐ND1s. a) Schematic model of ZF (zinc finger)‐ND1s. ZF‐ND1s, consisting of 6 assembled ZF proteins (shown in the green box with white numbering), amino acid linkers (shown in orange), and an ND1 heterodimer nuclease, are designed at the *AAVS‐1* locus with a 6‐bp spacer length (shown in red). A ZF protein recognizes 3 DNA bases (shown in green). b) T7E1 assay. A gel image of T7E1‐treated PCR products amplified from the human *AAVS‐1* site. The asterisks indicate a cleaved DNA band. c) Engineering of ZF‐ND1 using a 1‐bp skipping linker. The amino acid sequences are different between canonical and 1‐bp skipping linkers (underlined). A DNA base (shown in blue) is skipped by a 1‐bp skipping linker. d, e) T7E1 assay. A gel image of T7E1‐treated PCR products amplified from the target site. The asterisks indicate a cleaved DNA band. The transient cold shock was used in (e).

We designed ZF‐ND1 targeting human *AAVS‐1* (also known as *PPP1R12C*; chromosome 19) (**Figure** **1**a). In this design, an assembled ZF protein consisted of six fingers and recognized an 18 bp. Over two out of six fingers recognized the GNN. By linking left‐ and right‐assembled ZF proteins with ND1 RRR and DDD, respectively, we created a ZF‐ND1 pair. The spacer region was set to 6 bp. Consequently, the ZF‐ND1 pair recognized 36 bp and cleaved the target DNA in the 6‐bp spacer region. We created ten ZF‐ND1 pairs and transfected them into HEK293T cells. 48 h after transfection, genomic DNA was extracted and a T7 Endonuclease I (T7EI) assay was performed. The T7EI assay showed that two out of the 10 ZF‐ND1 pairs cleaved the target DNA (Figure 1b). We succeeded in obtaining two functional ZF‐ND1 pairs from the 10 ZF‐ND1 pairs tested. However, because the efficiency of genome editing is low (≈11%), methods for increasing it are required. Because ZF proteins easily recognize GNN,^[^ ^23^ ^]^ by increasing the number of fingers that recognize GNN using a 1‐bp skipping linker,^[^ ^24^ ^]^ we speculated that it might be possible to increase the efficiency of genome editing in this way. We first applied this approach to 9299L+R (#6), which has lower cleavage activity. Using 1‐bp skipping, we created 9299L (v2), in which four out of six fingers recognized the GNN (Figure 1c). We transfected the cells and performed the T7E1 assay at 48 h transfection. Highly enhanced genome editing at 18% was observed in the cells transfected with 9299L(v2)+R (Figure 1d). Similarly, we applied this approach to 2027L+R (#2) and created 2027L (v2), in which all six fingers recognized the GNN (Figure S1a, Supporting Information). No genome‐editing activity was observed in the cells transfected with 2027L(v2)+R (Figure S1b, Supporting Information). An approach that uses 1‐bp skipping linkers to increase the number of fingers that recognize the GNN may be effective. Because transient cold shock enhances ZFN‐mediated genome editing,^[^ ^25^ ^]^ we cultured the cells at 30 °C for 48 h after 24 h post‐transfection. Highly enhanced genome editing of 22, 16, and 26% was observed in the cells transfected with 2027L+R, 9299L+R, and 9299L(v2)+R, respectively (Figure 1e). Thus we can conclude that transient cold shock is a powerful method for enhancing genome editing. To detect the DNA cleavage activity at the reporter level, we performed a single‐strand annealing (SSA) assay (Figure S2a, Supporting Information). The SSA assay revealed that 2027L+R, 9299L+R, and 9299L(v2)+R exhibit DNA cleavage activity at the reporter level (Figure S2b, Supporting Information). To investigate whether functional ZF‐ND1s induce off‐target mutations, we selected the two highest potential off‐target sites of each ZF‐ND1 that were ranked using Nucleotide BLAST Identities. We amplified the targeted sites by PCR and subjected them to the T7E1 assay. No additional bands were detected between the non‐transfected and transfected cells (Figure S3a, Supporting Information), indicating that there were no mutations in the potential off‐target sites (Figure S3b, Supporting Information).

### 2.2 Structural Modeling of ZF‐ND1 Through AlphaFold, Coot, and Rosetta

**Figure 2:**
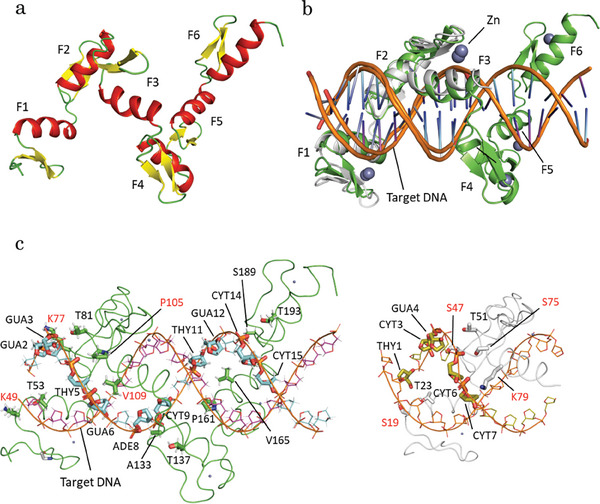
Model of a 2027R‐DNA complex. a) The ribbon model of the 2027R structure. b) A superimposed model of the 2027R‐DNA model and a crystal structure of Zif268‐DNA (PDB ID 1ZAA). The 2027R‐DNA model and the Zif268‐DNA structure are shown as ribbon and cartoon models, respectively. 2027R and Zif268 are colored green and white, respectively. The DNA structures of 2027R‐DNA and Zif268‐DNA are colored magenta, cyan, and white, respectively. The zinc atoms of both 2027R and Zif268 are indicated as grey sphere models. c) Structural comparison of the 2027R‐DNA model and the crystal structure of Zif268‐DNA. Putative interaction sites of 2027R (green) and the opposite‐chain DNA backbone (cyan) against its target DNA (magenta) with bold stick models and their labels. K49 (finger‐1; F1), K77 (finger‐2; F2), P105, and V109 (finger‐3; F3) in 2027R, located at 1 or 5 in the −1–6 recognition sites, are the same orientation as S19 (F1), S47 (F2), S75 and K79 (F3) in Zif268‐DNA, respectively. The residues mentioned above are shown in red text.

However, this synthetic biology approach has certain limitations. We could not increase the number of fingers that recognized the GNN by 2027R using a 1‐bp skipping linker. Other approaches are required to improve genome editing efficiency. Therefore, we attempted to design the structure interface of 2027R‐DNA using AlphaFold and RosettaDNA in the Rosetta application. First, 2027R (S32‐S172) containing canonical linkers (TGGS amino acid residues) was modeled using AlphaFold (**Figure** **2**a). The pLDDT and pTM score model qualities of 2027R were 93.54 and 0.6735, respectively. After modeling the double‐stranded DNA (18‐bp) by Coot, overlaying both 2027R and the DNA model with the Zif268‐DNA structure (PDB ID IZAA), resulting in a rmsd of 1.05‐Å, were similar (Figure 2b). A comparison of these structures and amino acid alignments showed that the amino acid residues at positions 1 and 5 of each ZF module, located near the phosphate backbone of the DNA, were different. Zif268 has three Ser residues (S19, S47, and S75) at position 1 and two Thr residues (T23 and T51) and one Lys residue (K79) at position 5 (Figure 2c; Table S4, Supporting Information). In contrast, 2027R had only one Ser residue at position 1 (amino acid residues K49, K77, P105, A133, P161, and S189) and no Lys residues at position 5 (T53, T81, V109, T137, V165, and T193) (Figure 2c; Table S4, Supporting Information). Therefore, we focused on positions 1 and 5 of 2027R, especially the eight residues (K49, K77, P105, V109, A133, P161, V165, and T193) that differed from those of Zif268 and modified them using RosettaDNA.

### 2.3 Engineering of ZF‐ND1 Based on Structural Modeling

**Figure 3:**
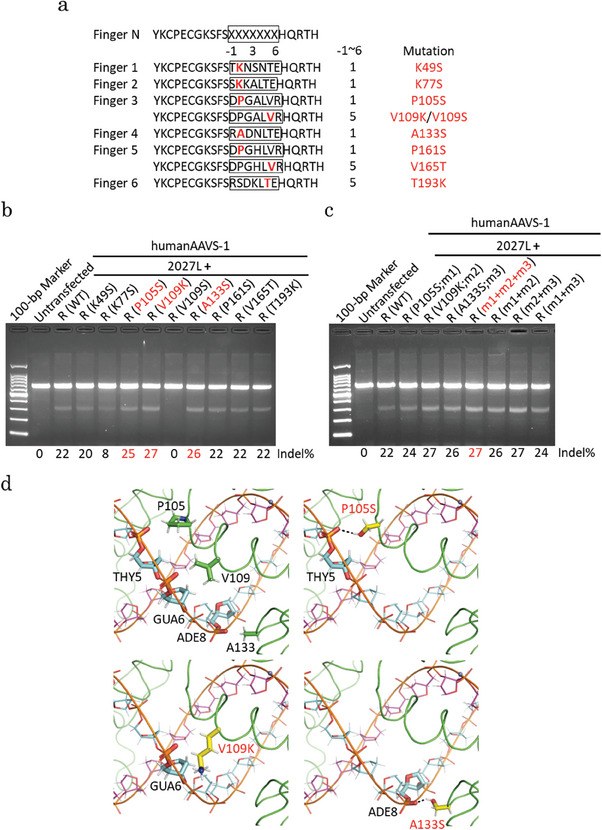
Engineering of ZF‐ND1 based on structural modeling. a) Amino acid sequences of each ZF protein in 2027R. Seven amino acids (‐1–6, surrounded by a square) are different among the ZF proteins. An amino acid (shown in bold red) at position 1 or 5 is changed to another amino acid (shown in red). b, c) T7E1 assay. A gel image of T7E1‐treated PCR products amplified from the human *AAVS‐1* site. P105S, V109K, and A133S are abbreviated as m1, m2, and m3, respectively in (c). d) Mutant models of 2027R with DNA. P105S, V109K, and A133S in 2027R, which are involved in improving ZF‐ND1 activity, are shown as bold stick models (yellow). Putative binding residues of the opposite chain of the DNA are shown as bold stick models (cyan). The back dashed lines represent putative hydrogen bonds.

ZF proteins’ α‐helices interact with the DNA major groove where each finger interacts with 3 DNA bases at the ‐1, 3, and 6 amino acid residue positions respectively.^[^ ^22^ ^]^ The mutation candidates that emerged from the structural modeling were K49S, K77S, P105S, V109K, V109S, A133S, P161S, V165T, and T193K, which are located at the 1 or 5 amino acid residue position (**Figure** **3**a). In RosettaDNA, the mutation candidates were selected based on the lowest energy (total score calculated as Rosetta Energy Unit (REU)) of the 2027R‐DNA complex structure after replacing each residue with almost all hydrophilic amino acids, except Cys and Tyr residues. Positions 1 and 5 of the α‐helix made direct or water‐mediated contact with the phosphate backbone of the DNA.^[^ ^26^ ^]^ To confirm whether that feature was optimized, mutation candidates were subjected to a cell‐based assay. We transfected mutated 2027R and 2027L into the cells and cultured them at 30 °C for 48 h after 24 h post‐transfection. In comparisons with wild‐type 2027R (WT), highly enhanced genome editing at 25, 27, or 26% was observed in the cells transfected with mutated 2027R (P105S, V109K, or A133S, respectively) and 2027L (Figure 3b). Furthermore, we introduced mutations that improved the genome editing efficiency in 2027R. No further increases were observed in genome editing efficiency (Figure 3c), indicating that multiple mutations were not effective. The V109K mutant at position 5 of finger‐3 in 2027R, substituted by the resifile‐directed design of the RosettaDNA application, was placed between the phosphate backbones of GUA6 and GUA7. Therefore, V109K may not directly interact with the phosphate backbones of GUA6 and GUA7 (Figure 3d). However, the total score of the 2027R V109K‐DNA complex, which means the total energy (REU) of the complex structure and an index of the structure stability in Rosetta, was lower than that of the WT‐DNA complex, suggesting that it was more stable (Figure S4, Supporting Information). On the other hand, the total score of the 2027R V109S‐DNA complex was lower than that of V109K, suggesting that the structure was stable (Figure S4, Supporting Information). However, the genome editing efficiency of the V109S mutant was completely lost (Figure 3b). P105S at position 1 of finger‐3 and A133S at position 1 of finger‐4 in 2027R, which improved genome editing efficiency, were suggested to form new hydrogen bonds with the phosphate backbones of THY5 and ADE8, respectively (Figure 3d). All the other mutants had lower total scores than the WT but had the same or lower genome editing efficiency (Figure 3b). We performed an SSA assay to detect DNA cleavage activity at the reporter level. The SSA assay indicated that the 2027L+mutated 2027R (P105S, V109K, or A133S) had DNA cleavage activity at the reporter level (Figure S5, Supporting Information). To investigate whether mutated ZF‐ND1s induce off‐target mutations, we amplified the off‐target candidate sites using PCR and subjected them to a T7E1 assay. No additional bands were detected between the non‐transfected and transfected cells (Figure S6a, Supporting Information), indicating that there were no mutations in the potential off‐target sites (Figure S6b, Supporting Information).

## 3 Discussion

Herein we developed functional ZF‐ND1 pairs and successfully improved their genome editing efficiency using a structural modeling approach. The results of the present study demonstrate that the modular assembly system is useful for creating 6‐finger ZF‐ND1s and that AlphaFold‐, Coot‐, and Rosetta‐based modeling is effective in improving its function.

Prior to this study, several studies have been conducted on the yield of ZFN‐based genome editing. However, no study has reported the efficient construction of functional ZF pairs using a modular assembly system and further improvements in its function. A previously reported modular assembly system was used for 3‐finger ZFNs. In this study, the system was used for 6‐finger ZFNs and was effective in creating functional ZFNs. This system may be more suitable for creating 6‐finger ZFNs than 3‐finger ZFNs. This study is the first to demonstrate the high functionalization of ZFNs through structural modeling using AlphaFold, Coot, and Rosetta. Because positions 1 and 5 of the α‐helix make contact with the phosphate backbone of the DNA, engineering the 1 or 5 amino acid residue position might be effective in improving ZFN function. In the Rosetta‐based 2027R design, the total scores (REU) of all the mutants at amino acid residues 1 or 5 in 2027R were lower than those in the WT, suggesting higher structural stability of the complexes (Figure S4, Supporting Information). Interestingly, among these mutants, P105S, V109K, and A133S, which were predicted to have high structural stability, showed increased genome editing efficiency (Figure 3b). P105S and V109K at finger‐3 of 2027R are also conserved in Zif268, suggesting that they are essential for recognizing the phosphate backbone of DNA in C_2_H_2_‐type ZFs. However, in this study, we successfully demonstrated that A133S in finger‐4 of 2027R, which is not present in Zif268, improved genome editing efficiency. Regarding the complex structure of more than four ZFs and DNA, a multidomain CCCTC‐binding factor (CTCF) belonging to the same C_2_H_2_‐type ZF family and containing a tandem array of 11 ZFs has been reported (PDB ID 5UND).^[^ ^27^ ^]^ Intriguingly, position 1 of finger‐4 in CTCF is not Ser but Lys, forming a hydrogen bond with the phosphate backbone of DNA (Figure S7a, Supporting Information). In CTCF, the linker between finger‐3 and finger‐4, and the α‐helix of finger‐3 are longer than 2027R, and the conformation of the α‐helix of finger‐4 is different (Figure S7, Supporting Information). Therefore, a large polar amino acid such as Lys would be more suitable for binding the α‐helix of finger‐4 to the phosphate backbone of the DNA. In contrast, in 2027R, the distance between A133 and the phosphate backbone of the DNA is relatively small, suggesting that Ser, a small polar residue, would be selected by RosettaDNA (Figure S7b, Supporting Information). Thus, the combination of AlphaFold modeling and Rosetta DNA‐based protein design methods could be a promising tool to improve genome‐editing efficiency.

To clarify whether the V109K mutation increased genome editing efficiency in different DNA sequences, we engineered other ZF‐ND1s. T7E1 assay showed that the V109K mutation increased the efficiency of genome editing at different sites by 3–5% (Figure S8, Supporting Information).

Our results indicated that the engineering of ZFNs using structural modeling increased efficiency by 5%, from 22 to 27%. However, this increase in genome editing efficiency was still low, and, thus, other factors, such as binding energy and conformational changes, need to be considered in addition to structural stability when modeling in future studies. Moreover, since ZF proteins interact with 3 DNA bases at the ‐1, 3, and 6 amino acid residue positions,^[^ ^22^ ^]^ it may be possible to achieve further improvements in ZFN by engineering amino acid residues at positions ‐1, 3, and 6 based on structural modeling.

In the field of ophthalmology, genome editing is a promising tool for the treatment of genetic disorders. Retinitis pigmentosa (RP) is an inherited photoreceptor degeneration disease.^[^ ^28^ ^]^ More than 200 mutations have been identified in the RP disease gene *RHO*.^[^ ^29^ ^]^ Mutation‐independent gene knock‐in therapy targeting the 5’ untranslated region (UTR) was previously shown to be an effective treatment for *RHO*‐associated RP.^[^ ^30^ ^]^ To achieve efficient gene knock‐in at 5’UTR, a highly efficient genome editing tool is required. ZFNs that increase the efficiency of genome editing are useful for this purpose. By combining engineered ZFNs with an AAV vector, it may be possible to achieve highly efficient genome editing and gene knock‐in in the retina.

ZFNs are smaller in size than TALENs and CRISPR‐Cas9. Therefore, ZFN‐encoding DNAs can be easily packed into an AAV vector with a limited cargo space. AAV/ZFN provides a more powerful platform for translational research and clinical use. ZFNs have potential applications in epigenetic editing. Instead of using a Cas9‐based system for editing DNA methylation,^[^ ^31^ ^]^ a ZFN‐based system for editing it could be achieved. Unlike for CRISPR‐Cas9, the patents for ZFNs have already expired; thus, high patent royalties are not required for industrial applications.

In summary, the efficient construction of functional ZF‐ND1s and improvements in their functions provide a promising platform for various industrial applications without high patent royalties.

## 4 Experimental Section

### ZF Design

The Zinc Finger Tool (http://www.zincfingertools.org/)^[^ ^14c^ ^]^ was used to design ZFs for the target site. Among Toolgen,^[^ ^32^ ^]^ Sangamo,^[^ ^24^ ^]^ and the Barbas module,^[^ ^33^ ^]^ the Barbas module was used. The Zinc Finger Tool was incorporated only with the Barbas module. The designed ZFs are shown in Table S1 (Supporting Information).

### Vector Construction

An assembled ZF was constructed using the Barbas module (kindly provided by Dr. Wataru Nomura, deposited in Addgene as ZF58–106). ZF fragments were amplified from the ZF58–106 plasmids using the KOD One PCR Master Mix (Toyobo) and the appropriate primer sets (Table S2, Supporting Information). Golden gate assembly was used to assemble the ZF fragments. Assembled ZF fragments were cloned into ND1RRR or ND1DDD expression vectors by XbaI and BamHI double digestion. NEB Stable Competent Cells (NEB) were used for plasmid transformation and the plasmids were purified using the QIAprep Spin Miniprep Kit (QIAGEN) according to the manufacturer's instructions. The nucleotide sequences were validated using Sanger sequencing. The SSA reporter plasmid^[^ ^34^ ^]^ was constructed by inserting annealed primers (Table S2, Supporting Information). The nucleotide sequences were validated using Sanger sequencing.

### Cell Culture and DNA Transfection

HEK293T cells were cultured at 37 °C under 5% CO_2_ in Dulbecco's Modified Eagle Medium (DMEM, Nacalai Tesque) supplemented with 10% FBS (HyClone), 100 unit mL^−1^ of penicillin, and 100 µg mL^−1^ of streptomycin (Nacalai Tesque). Lipofectamine 2000 (Life Technologies) and Opti‐MEM (Life Technologies) for HEK293T were used for the transfection, according to the manufacturer's instructions. The plasmid concentrations, cell numbers, and plates used were as follows: 150 ng of the left ZF‐ND1RRR and right ZF‐ND1DDD plasmids (300 ng total) were added to 0.5 × 10^5^ cells in a 24‐well plate.

In the SSA assay, the plasmid concentrations, cell numbers, and plates used were as follows: 150 ng of the left ZF‐ND1RRR and right ZF‐ND1DDD plasmids and 100 ng of SSA reporter plasmids (400 ng in total) into 0.5 × 10^5^ cells using a 24‐well plate.

### Transient Cold Shock

At 24 h following transfection, the culture temperature was changed to 30 °C. The cells were additionally cultured at 30 °C for 48 h. At 72 h following transfection, the cells were harvested and subjected to a T7EI assay.

### T7E1 Assay

Genomic DNA was extracted using a QIAamp DNA Mini Kit (QIAGEN) prior to the PCR. The target site was amplified with the KOD One PCR Master Mix (Toyobo) using the appropriate primer set (Table S3, Supporting Information). The PCR products were purified using a MinElute PCR Purification Kit (QIAGEN). Following this, denaturing and reannealing reactions were conducted at 95 °C for 5 min, 95 to 85 °C at −2.0 °C s^−1^, 85 to 25 °C at −0.1 °C sec^−1^, and finally 4 °C for ∞. The heteroduplexed PCR products were incubated with T7E1 (NEB) at 37 °C for 30 min. The resulting products were analyzed by electrophoresis on a 2% agarose gel and visualized using Gel Red. The intensities of the bands of the PCR amplicon and cleavage products were measured using ImageJ (NIH). The efficiency was calculated using the following formula: % gene modification = 100 × (1 − (1 − (b+c)/(a+b+c))^1/2^), where a is the intensity of the undigested PCR product, and b and c are the intensities of each cleavage product.

### Off‐Target Analyses

Nucleotide BLAST (https://blast.ncbi.nlm.nih.gov/Blast.cgi) was used to identify off‐target candidate sites for the ZF‐ND1 pairs. The PCR‐amplified candidate sites were evaluated using the T7E1 assay. The primers used are listed in Table S3 (Supporting Information).

### Flow Cytometry

At 48 h following transfection, the cells were harvested using 0.25% trypsin‐EDTA and washed with the culture medium. After washing, the cells were suspended in a FACS buffer (PBS, 5% FBS, and 2 mM EDTA). Flow cytometric analysis was performed on a Cell Sorter MA900 machine (SONY) using wavelength analysis (488 nm solid‐state laser).

### Key ZF‐ND1 Amino Acid Sequences

ZF, linker, and ND1 are shown in green, orange, and blue, respectively.

2027L‐ND1RRR

MGPKKKRKVAAAPAAKRVKLDGSGPKKKRKVSRPKPYKCPECGKSFSRNDALTEHQRTHTGEKPYKCPECGKSFSRSDDLVRHQRTHTGQKPYKCPECGKSFSRSDKLVRHQRTHTGEKPYKCPECGKSFSRSDKLTEHQRTHTGQKPYKCPECGKSFSRSDKLVRHQRTHTGEKPYKCPECGKSFSDCRDLARHQRTHTGGSLVKGEMEKKKSDLRHKLKHVPHEYIELIEIAQDSKQNRLFEFKVVEFLKEVYDYNGKHLGGSRKPDGALYTNGLKTDYGIILDTKAYKDGYSLPISQAREMQRYVDENNNRNAIINPNEWWKVYPNSILDFKFLFVSGFFKGDYKKQLARVSRLTKRKGAVLSVEQLLLGGEKIKDGSLTLEDVGDKFNNDEIIF

2027R‐ND1DDD

MGPKKKRKVAAAPAAKRVKLDGSGPKKKRKVSRPKPYKCPECGKSFSTKNSLTEHQRTHTGEKPYKCPECGKSFSSKKALTEHQRTHTGQKPYKCPECGKSFSDPGALVRHQRTHTGEKPYKCPECGKSFSRADNLTEHQRTHTGQKPYKCPECGKSFSDPGHLVRHQRTHTGEKPYKCPECGKSFSRSDKLTEHQRTHTGGSLVKGEMEKKKSDLRHKLKHVPHEYIELIEIAQDSKQNRLFEFKVVEFLKEVYDYNGKHLGGSRKPDGALYTNGLKTDYGIILDTKAYKDGYSLPISQADEMQDYVDENNNRDAIINPNEWWKVYPNSILDFKFLFVSGFFKGDYKKQLARVSNLTKRKGAVLSVEQLLLGGEKIKDGSLTLEDVGDKFNNDEIIF

9299L‐ND1RRR

MGPKKKRKVAAAPAAKRVKLDGSGPKKKRKVSRPKPYKCPECGKSFSRSDKLTEHQRTHTGEKPYKCPECGKSFSQSGNLTEHQRTHTGQKPYKCPECGKSFSRSDHLTTHQRTHTGEKPYKCPECGKSFSDPGNLVRHQRTHTGQKPYKCPECGKSFSQSGHLTEHQRTHTGEKPYKCPECGKSFSTSGNLVRHQRTHTGGSLVKGEMEKKKSDLRHKLKHVPHEYIELIEIAQDSKQNRLFEFKVVEFLKEVYDYNGKHLGGSRKPDGALYTNGLKTDYGIILDTKAYKDGYSLPISQAREMQRYVDENNNRNAIINPNEWWKVYPNSILDFKFLFVSGFFKGDYKKQLARVSRLTKRKGAVLSVEQLLLGGEKIKDGSLTLEDVGDKFNNDEIIF

9299R‐ND1DDD

MGPKKKRKVAAAPAAKRVKLDGSGPKKKRKVSRPKPYKCPECGKSFSRSDKLVRHQRTHTGEKPYKCPECGKSFSTKNSLTEHQRTHTGQKPYKCPECGKSFSDPGHLVRHQRTHTGEKPYKCPECGKSFSTKNSLTEHQRTHTGQKPYKCPECGKSFSTSGSLVRHQRTHTGEKPYKCPECGKSFSDPGALVRHQRTHTGGSLVKGEMEKKKSDLRHKLKHVPHEYIELIEIAQDSKQNRLFEFKVVEFLKEVYDYNGKHLGGSRKPDGALYTNGLKTDYGIILDTKAYKDGYSLPISQADEMQDYVDENNNRDAIINPNEWWKVYPNSILDFKFLFVSGFFKGDYKKQLARVSNLTKRKGAVLSVEQLLLGGEKIKDGSLTLEDVGDKFNNDEIIF

9299L(v2)‐ND1RRR

MGPKKKRKVAAAPAAKRVKLDGSGPKKKRKVSRPKPYKCPECGKSFSRSDKLVRHQRTHTGEKPYKCPECGKSFSDSGNLRVHQRTHTGQKPYKCPECGKSFSDPGHLVRHQRTHTHPRAPIPKPYKCPECGKSFSDPGNLVRHQRTHTGQKPYKCPECGKSFSQSGHLTEHQRTHTGEKPYKCPECGKSFSTSGNLVRHQRTHTGGSLVKGEMEKKKSDLRHKLKHVPHEYIELIEIAQDSKQNRLFEFKVVEFLKEVYDYNGKHLGGSRKPDGALYTNGLKTDYGIILDTKAYKDGYSLPISQAREMQRYVDENNNRNAIINPNEWWKVYPNSILDFKFLFVSGFFKGDYKKQLARVSRLTKRKGAVLSVEQLLLGGEKIKDGSLTLEDVGDKFNNDEIIF

2027R(P105S)‐ND1DDD

MGPKKKRKVAAAPAAKRVKLDGSGPKKKRKVSRPKPYKCPECGKSFSTKNSLTEHQRTHTGEKPYKCPECGKSFSSKKALTEHQRTHTGQKPYKCPECGKSFSDSGALVRHQRTHTGEKPYKCPECGKSFSRADNLTEHQRTHTGQKPYKCPECGKSFSDPGHLVRHQRTHTGEKPYKCPECGKSFSRSDKLTEHQRTHTGGSLVKGEMEKKKSDLRHKLKHVPHEYIELIEIAQDSKQNRLFEFKVVEFLKEVYDYNGKHLGGSRKPDGALYTNGLKTDYGIILDTKAYKDGYSLPISQADEMQDYVDENNNRDAIINPNEWWKVYPNSILDFKFLFVSGFFKGDYKKQLARVSNLTKRKGAVLSVEQLLLGGEKIKDGSLTLEDVGDKFNNDEIIF

2027R(V109K)‐ND1DDD

MGPKKKRKVAAAPAAKRVKLDGSGPKKKRKVSRPKPYKCPECGKSFSTKNSLTEHQRTHTGEKPYKCPECGKSFSSKKALTEHQRTHTGQKPYKCPECGKSFSDPGALKRHQRTHTGEKPYKCPECGKSFSRADNLTEHQRTHTGQKPYKCPECGKSFSDPGHLVRHQRTHTGEKPYKCPECGKSFSRSDKLTEHQRTHTGGSLVKGEMEKKKSDLRHKLKHVPHEYIELIEIAQDSKQNRLFEFKVVEFLKEVYDYNGKHLGGSRKPDGALYTNGLKTDYGIILDTKAYKDGYSLPISQADEMQDYVDENNNRDAIINPNEWWKVYPNSILDFKFLFVSGFFKGDYKKQLARVSNLTKRKGAVLSVEQLLLGGEKIKDGSLTLEDVGDKFNNDEIIF

2027R(A133S)‐ND1DDD

MGPKKKRKVAAAPAAKRVKLDGSGPKKKRKVSRPKPYKCPECGKSFSTKNSLTEHQRTHTGEKPYKCPECGKSFSSKKALTEHQRTHTGQKPYKCPECGKSFSDPGALVRHQRTHTGEKPYKCPECGKSFSRSDNLTEHQRTHTGQKPYKCPECGKSFSDPGHLVRHQRTHTGEKPYKCPECGKSFSRSDKLTEHQRTHTGGSLVKGEMEKKKSDLRHKLKHVPHEYIELIEIAQDSKQNRLFEFKVVEFLKEVYDYNGKHLGGSRKPDGALYTNGLKTDYGIILDTKAYKDGYSLPISQADEMQDYVDENNNRDAIINPNEWWKVYPNSILDFKFLFVSGFFKGDYKKQLARVSNLTKRKGAVLSVEQLLLGGEKIKDGSLTLEDVGDKFNNDEIIF

### Modeling of Protein‐DNA Interactions

2027R (S32‐S172) containing a canonical linker (TGGS amino acid residues) and its target double‐stranded DNA (18‐bp) of human *AAVS‐1* were modeled using AlphaFold^[^ ^18^ ^]^ and COOT applications,^[^ ^19^ ^]^ respectively. An initial model of the 2027R−DNA complex was created by superimposition with the Zif268−DNA complex (PDB ID: 1ZAA). Next, the 2027R (including six Zn atoms)−DNA complex model was refined using the relaxing application of Rosetta,^[^ ^35^ ^]^ which searches the local conformational space around the initial structure. To improve the binding affinity of 2027R (positions 1 and 5 in ‐1–6 amino acid residues) to the opposite chain of the DNA, the RosettaDNA application^[^ ^21^ ^]^ using both a RosettaScripts XML‐like protocol file and a resfile was performed. The resfile specified only one of each of the eight amino acids (K49, K77, P105, V109, A133, P161, V165, and T193) at position 1 or 5 of the amino acid residues during the RosettaDNA run and introduced mutations using the PIKAA command. For each mutant of 2027R, including the WT, 1000 models of the 2027R−DNA complex were sampled.

After running RosettaDNA, the top 10 2027R‐DNA complex models with the total score for each mutant were extracted. All the figures were generated using the open‐source PyMol 2.4 software (PyMOL Molecular Graphics System, v2.4 Schrödinger, LLC, http://www.pymol.org/).^[^ ^36^ ^]^

## Author Contributions

S. K. and T. Y. conceived and designed the study. S. K. performed the experiments. M.W. performed the structural modeling. S. K., M. W., Y. K., W. N., and T. Y. analyzed the data. S. K., M. W., and T. Y. wrote the manuscript. All the authors discussed the results.

## Conflict of Interest

The authors declare no conflict of interest.

## Supporting information

Supporting Information

## Data Availability

The data that support the findings of this study are available in the supplementary material of this article
