# Supplementary material for: Engineering of Zinc Finger Nucleases Through Structural Modeling Improves Genome Editing Efficiency in Cells
Source: Adv Sci (Weinh). 2024 Apr 10;11(23):2310255. doi: 10.1002/advs.202310255 (PMC11187957; doi:10.1002/advs.202310255)
Supplement: Supplementary file 1 — Supporting Information [file ADVS-11-2310255-s001.pdf]

ADVANCED SCIENCE
Open Access

Supporting Information

for *Adv. Sci.*, DOI 10.1002/advs.202310255

Engineering of Zinc Finger Nucleases Through Structural Modeling Improves Genome Editing Efficiency in Cells

Shota Katayama, Masahiro Watanabe, Yoshio Kato, Wataru Nomura and Takashi Yamamoto**

Figure S1 (Katayama et al.)

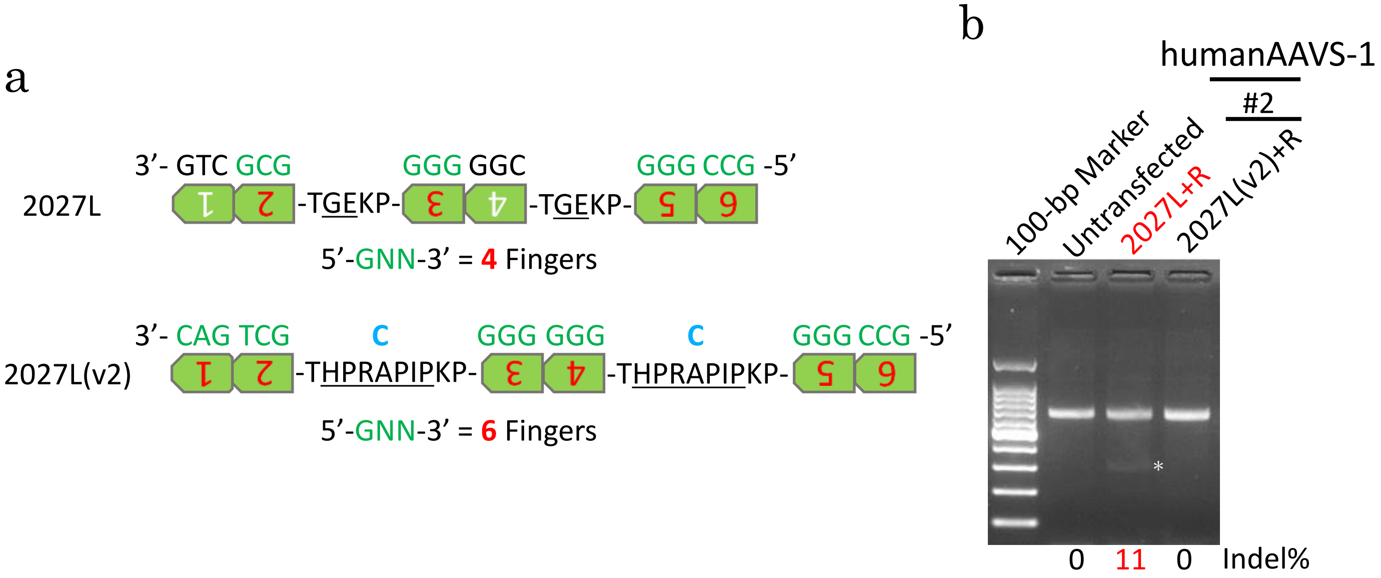

Figure S1. Engineering of ZF-ND1 using a 1-bp skipping linker. (**a**) Schematic model of the engineering of ZF-ND1 using a 1-bp skipping linker. A DNA base (shown in blue) is skipped by the 1-bp skipping linker. (**b**) T7E1 assay. A gel image of T7E1-treated PCR products amplified from the human *AAVS-1* site. The asterisk indicates a cleaved DNA band.

Figure S2 (Katayama et al.)

a

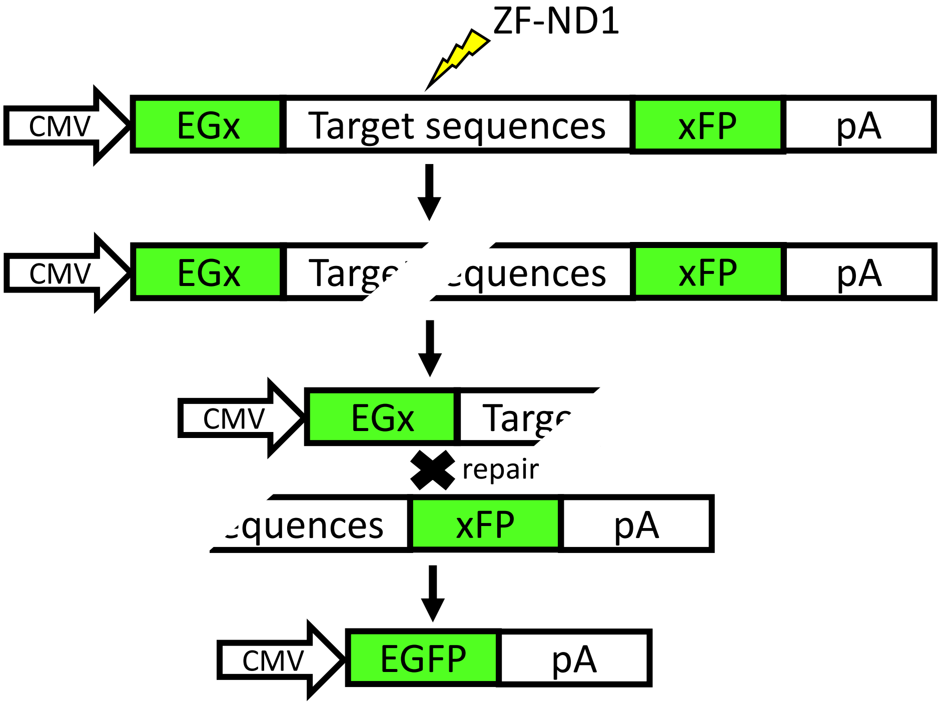

b

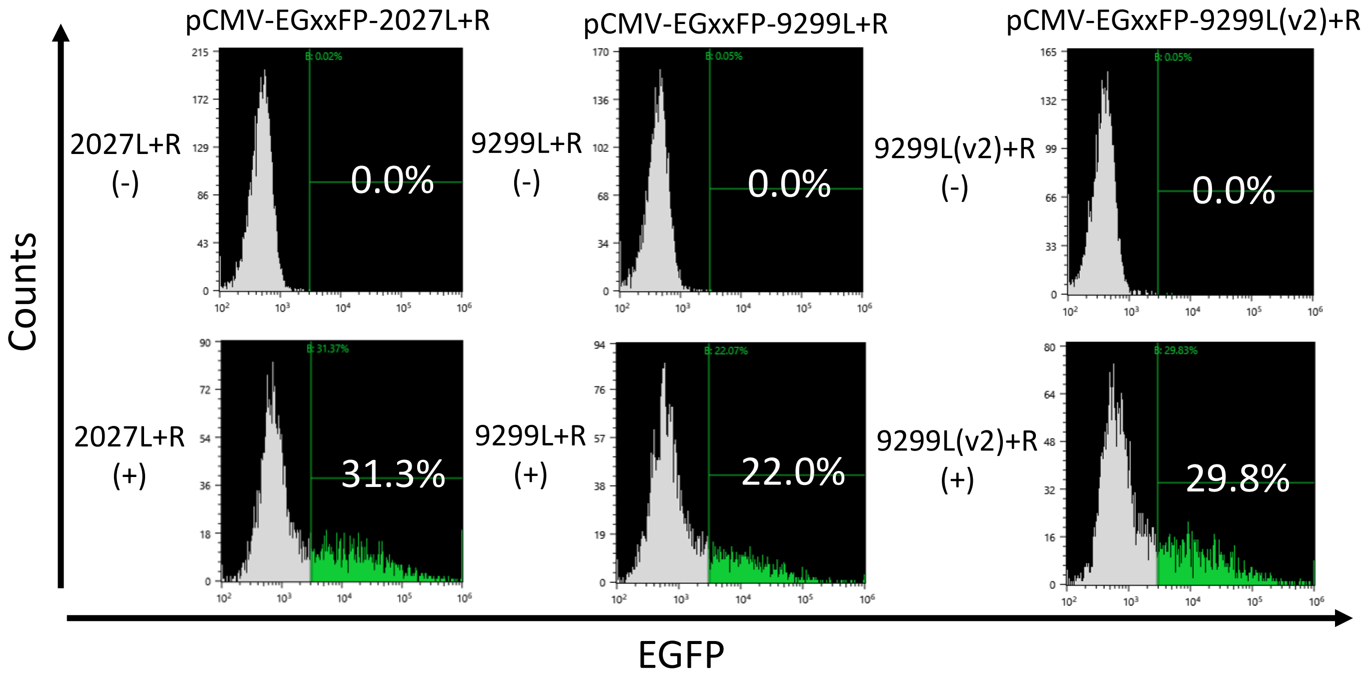

Figure S2. SSA assay. (**a**) Reconstitution of *EGFP* through ZF/ND1 mediated a homology-directed repair (HDR). HDR results in the expression of *EGFP*. (**b**) FACS analysis of the EGFP expression. HEK293T cells, which were transfected with pCMV-EGxxFP-2027L+R and ZF-ND1 (left panels), -9299L+R and ZF-ND1 (middle panels), or -9299L(v2)+R and ZF-ND1 (right panels), were analyzed at 48 hours post-transfection by flow cytometry.

Figure S3 (Katayama et al.)

a

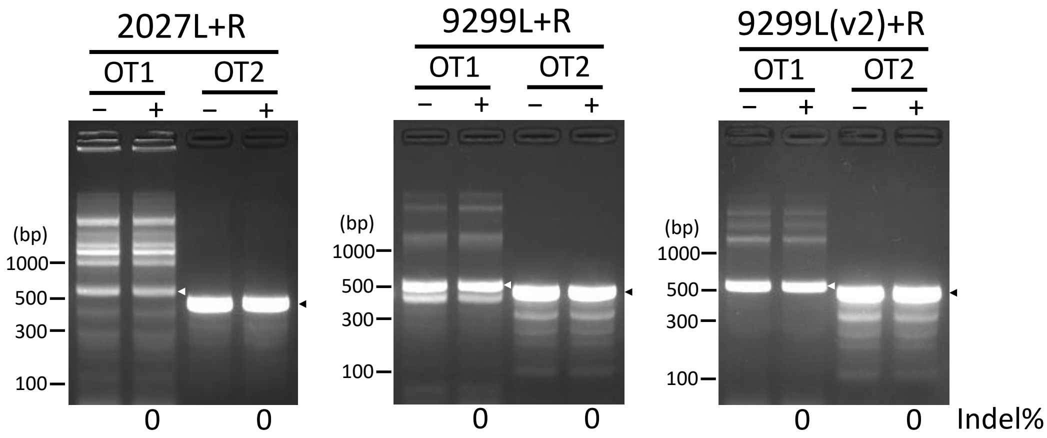

b

Target gene	Prototype	Name	Sequence (Top strand: 5' to 3', Bottom strand: 3' to 5')	Coordinate	Genomic region	Mutation	Identities (%)
Human AAVS-1	2027L+R	On-target	CAG CGC CCC CCG CCC GGC GTCTCC CGG GGC CAG GTC CAC CCT<br>GTC GCG GGG GGC GGG CCG CAGAGG GCC CCG GTC CAG GTG GGA	Chr19: 55117179-55117220	Intron	-	-
		OT1	CAG CGC CCC CCG CCC GGC CGCCGC TCG GAG CCA CCG CCC GCC<br>GTC GCG GGG GGC GGG CCG GCGGCG AGC CTC GGT GGC GGG CGG	Chr3: 46882282-46882323	Intergenic	Not detected	66.7
		OT2	TCT GGA GGA CGG GGA GGC AGCCCC AGG GGC CAG GTC CAC CCT<br>AGA CCT CCT GCC CCT CCG TCGGGG TCC CCG GTC CAG GTG GGA	Chr8: 143876342-143876383	Intron	Not detected	63.9
	9299L+R	On-target	CCG TTG CCA GTC TCG ATC CGCCCC GTC GTT CCT GGC CCT GGG<br>GGC AAC GGT CAG AGC TAG GCGGGG CAG CAA GGA CCG GGA CCC	Chr19: 55117107-55117148	Intron	-	-
		OT1	CCG TTG CCA GTC TCG TTG GGAGAT CAA AAA CCA TCG TTC AGC<br>GGC AAC GGT CAG AGC AAG CCTCTA GTT TTT GGT AGC AAG TCG	Chr1: 39300244-39300285	Exon	Not detected	55.6
		OT2	GCA TAG GAT TGC CAA GTA CCAGGA CTC GTT CCT GGC CCT GGA<br>CGT ATC CTA ACG GTT CAT GGTCCT GAG CAA GGA CCG GGA CCT	Chr12: 78280388-782803429	Intergenic	Not detected	58.3
	9299Lv2+R	On-target	CCC GTT GCC GTC TCG ATG CGCCCC GTC GTT CCT GGC CCT GGG<br>GGG CAA CGG CAG AGC TAG GCGGGG CAG CAA GGA CCG GGA CCC	Chr19: 55117107-55117149	Intron	-	-
		OT1	GCC GTT GCC GTC TAG ATC CAGCCG ATA AAG GGC TGG GCT GGC<br>CGG CAA CGG CAG ATC TAG GTCGGC TAT TTC CCG ACC CGA CCG	Chr7: 28956967-28957008	Exon	Not detected	61.1
		OT2	GCA TAG GAT TGC CAA GTA CCAGGA CTC GTT CCT GGC CCT GGA<br>CGT ATC CTA ACG GTT CAT GGTCCT GAG CAA GGA CCG GGA CCT	Chr12: 78280388-782803429	Intergenic	Not detected	55.6

Figure S3. Off-target analysis of ZF-ND1s. (**a**) T7E1 assay. A gel image of T7E1-treated PCR products amplified from the target sites. The arrows indicate the corresponding band for the target site. OT1 and 2 indicate the off-target candidate sites 1 and 2, respectively. The cells untransfected (-) and transfected (+) with ZF-ND1 were subjected to a T7E1 assay. (**b**) Summary of an off-target analysis of ZF-ND1s. The red and blue letters indicate left and right ZF-ND1 target sequences, respectively. Mismatches are underlined. Identities were calculated by ((the number of match sequences) / 36) x 100.

Figure S4 (Katayama et al.)

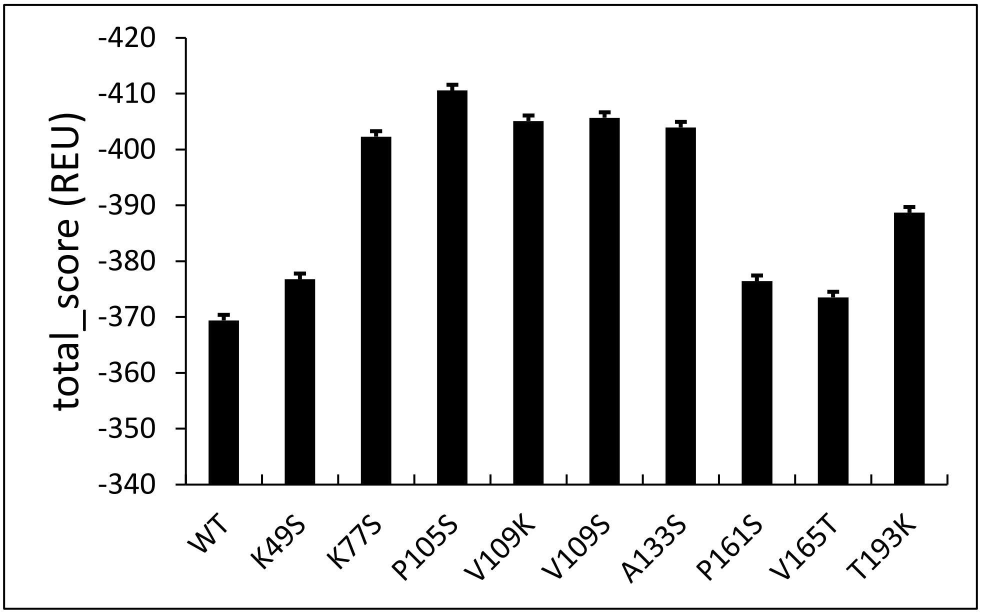

Figure S4. Total score (REU) of the WT and mutants of 2027R and DNA complexes. The mean of the total score of the top 10 models for each complex, as calculated by RosettaDNA, is shown. Standard errors (SE) are indicated by black bars.

Figure S5 (Katayama et al.)

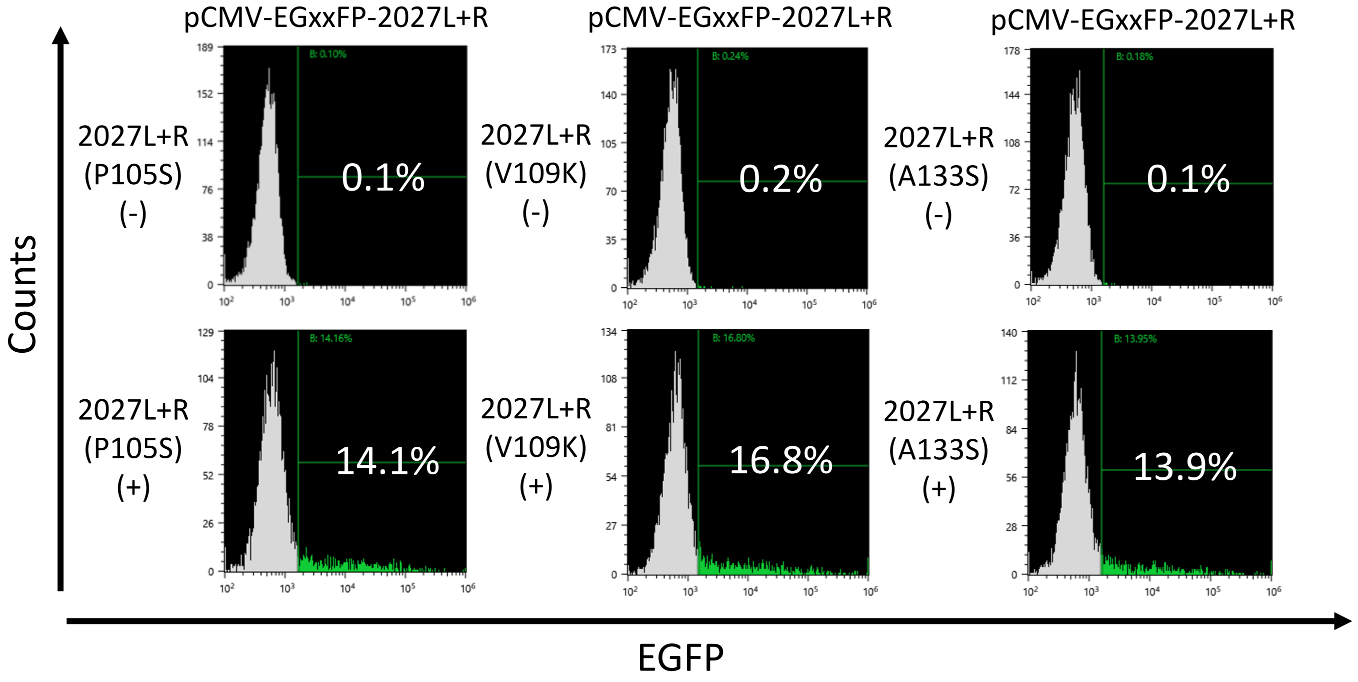

Figure S5. SSA assay. FACS analysis of the EGFP expression. HEK293T cells, which were transfected with pCMV-EGxxFP-2027L+R and 2027L+mutated 2027R (P105S; left panels, V109K; middle panels, A133S; right panels), were analyzed at 48 hours post-transfection by flow cytometry.

Figure S6 (Katayama et al.)

a

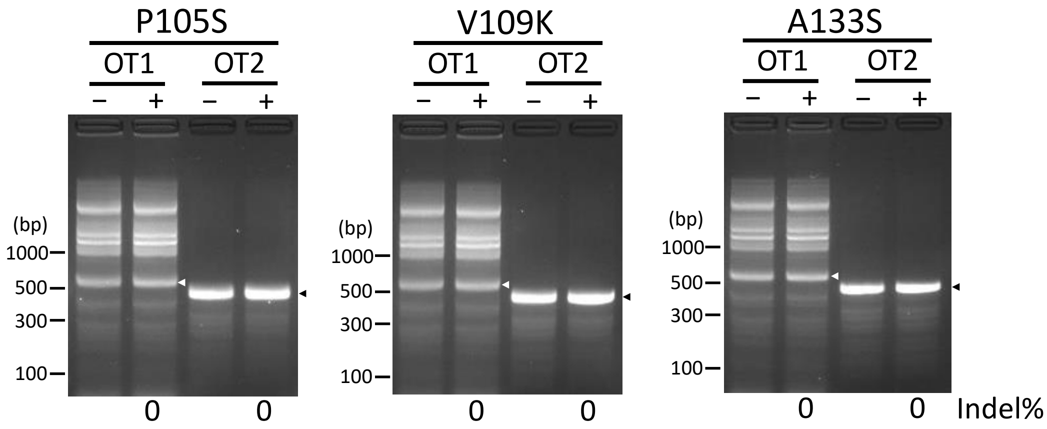

b

Target gene	Prototype	Name	Sequence (Top strand: 5' to 3', Bottom strand: 3' to 5')	Coordinate	Genomic region	Mutation	Identities (%)
		On-target	CAG CGC CCC CCG CCC GGC GTCTCC CGG GGC CAG GTC CAC CCT<br>GTC GCG GGG GGC GGG CCG CAGAGG GCC CCG GTC CAG GTG GGA	Chr19: 55117179-55117220	Intron	-	-
	2027L+R P105S	OT1	CAG CGC CCC CCG CCC GGC CGCCGC TCG GAG CCA CCG CCC GCC<br>GTC GCG GGG GGC GGG CCG GCGGCG AGC CTC GGT GGC GGG CGG	Chr3: 46882282-46882323	Intergenic	Not detected	66.7
		OT2	TCT GGA GGA CGG GGA GGC AGCCCC AGG GGC CAG GTC CAC CCT<br>AGA CCT CCT GCC CCT CCG TCGGGG TCC CCG GTC CAG GTG GGA	Chr8: 143876342-143876383	Intron	Not detected	63.9
		On-target	CAG CGC CCC CCG CCC GGC GTCTCC CGG GGC CAG GTC CAC CCT<br>GTC GCG GGG GGC GGG CCG CAGAGG GCC CCG GTC CAG GTG GGA	Chr19: 55117179-55117220	Intron	-	-
Human AAVS-1	2027L+R V109K	OT1	CAG CGC CCC CCG CCC GGC CGCCGC TCG GAG CCA CCG CCC GCC<br>GTC GCG GGG GGC GGG CCG GCGGCG AGC CTC GGT GGC GGG CGG	Chr3: 46882282-46882323	Intergenic	Not detected	66.7
		OT2	TCT GGA GGA CGG GGA GGC AGCCCC AGG GGC CAG GTC CAC CCT<br>AGA CCT CCT GCC CCT CCG TCGGGG TCC CCG GTC CAG GTG GGA	Chr8: 143876342-143876383	Intron	Not detected	63.9
		On-target	CAG CGC CCC CCG CCC GGC GTCTCC CGG GGC CAG GTC CAC CCT<br>GTC GCG GGG GGC GGG CCG CAGAGG GCC CCG GTC CAG GTG GGA	Chr19: 55117179-55117220	Intron	-	-
	2027L+R A133S	OT1	CAG CGC CCC CCG CCC GGC CGCCGC TCG GAG CCA CCG CCC GCC<br>GTC GCG GGG GGC GGG CCG GCGGCG AGC CTC GGT GGC GGG CGG	Chr3: 46882282-46882323	Intergenic	Not detected	66.7
		OT2	TCT GGA GGA CGG GGA GGC AGCCCC AGG GGC CAG GTC CAC CCT<br>AGA CCT CCT GCC CCT CCG TCGGGG TCC CCG GTC CAG GTG GGA	Chr8: 143876342-143876383	Intron	Not detected	63.9

Figure S6. Off-target analysis of ZF-ND1s. (**a**) T7E1 assay. A gel image of T7E1-treated PCR products amplified from the target sites. The arrows indicate the corresponding band for the target site. OT1 and 2 indicate the off-target candidate sites 1 and 2, respectively. The cells untransfected (-) and transfected (+) with ZF-ND1 were subjected to a T7E1 assay. (**b**) Summary of an off-target analysis of ZF-ND1s. The red and blue letters indicate left and right ZF-ND1 target sequences, respectively. Mismatches are underlined. Identities were calculated by ((the number of match sequences) / 36) x 100.

Figure S7 (Katayama et al.)

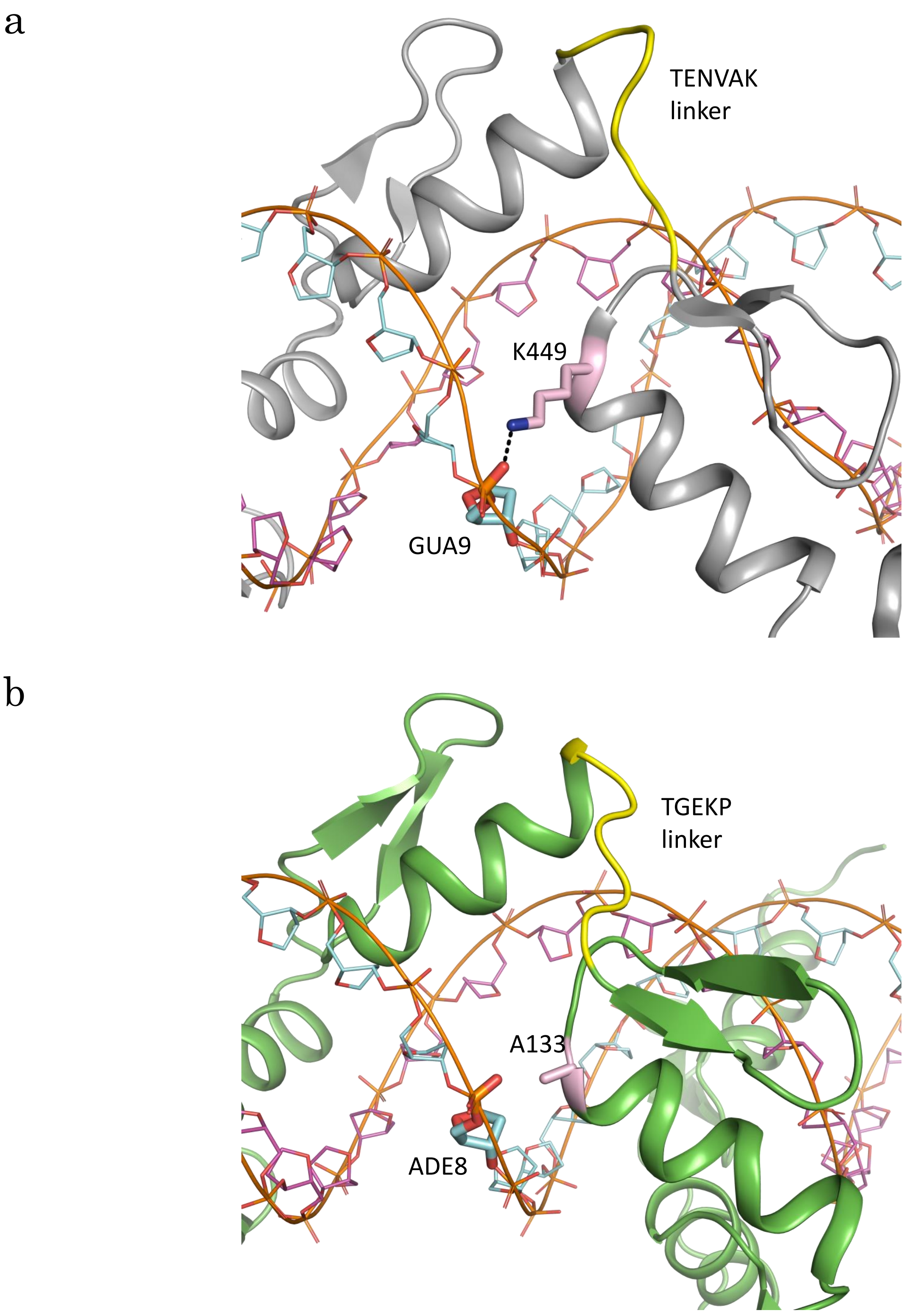

Figure S7. Comparison of position 1 of finger-4 in CTCF and 2027R. The complex structure of CTCF-DNA (**a**) and the model of 2027R-DNA (**b**). CTCF (grey), 2027R (green), and their DNA (cyan and magenta) are shown as ribbon and stick models. The TENVAK linker in CTCF and the TGEKP linker in 2027R, located between finger-3 and finger-4, are colored yellow. K449 (pink) of CTCF and GUA9 (cyan) and A133 (pink) of 2027R and ADE8 (cyan) are shown as fat stick models. A hydrogen bond is indicated by a black dashed line. The RMSD of both structures is 1.715 Å.

Figure S8 (Katayama et al.)

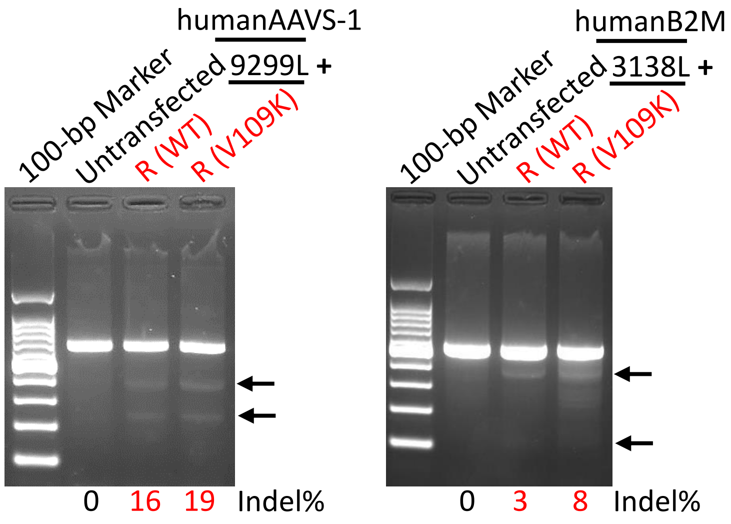

Figure S8. Engineering of ZF-ND1 using the V109K mutation. T7E1 assay. A gel image of T7E1-treated PCR products amplified from the human *AAVS-1* and *B2M* sites. Arrowheads indicate cleaved DNA bands.

Table S1. ZF designs for human *AAVS-1* and *B2M*

Prototype	Target sequences	Finger 1	Finger 2	Finger 3	Finger 4	Finger 5	Finger 6	Finger 1	Finger 2	Finger 3	Finger 4	Finger 5	Finger 6
1926L+R	5'-TCA GCG CCC CCC GCC CGG CGTCTC **CCG GGG CCA GGT CCA CCC**-3'	CCC	CCA	GGT	CCA	GGG	CCG	ZF94	ZF93	ZF60	ZF93	ZF58	ZF95
	3'-**AGT CGC GGG GGG CGG GCC** GCAGAG GGC CCC GGT CCA GGT GGG-5'	TGA	CGC	GGG	GGG	GGC	CCG	ZF105	ZF98	ZF58	ZF58	ZF61	ZF95
2027L+R	5'-CAG CGC CCC CCG CCC GGC GTCTCC **CGG GGC CAG GTC CAC CCT**-3'	CCT	CAC	GTC	CAG	GGC	CGG	ZF96	ZF90	ZF69	ZF91	ZF61	ZF99
	3'-**GTC GCG GGG GGC GGG CCG** CAGAGG GCC CCG GTC CAG GTG GGA-5'	CTG	GCG	GGG	CGG	GGG	GCC	ZF102	ZF70	ZF58	ZF99	ZF58	ZF73
2330L+R	5'-CGC CCC CCG CCC GGC GTC TCCCGG **GGC CAG GTC CAC CCT CTG**-3'	CTG	CCT	CAC	GTC	CAG	GGC	ZF102	ZF96	ZF90	ZF69	ZF91	ZF61
	3'-**GCG GGG GGC GGG CCG CAG** AGGGCC CCG GTC CAG GTG GGA GAC-5'	GCG	GGG	CGG	GGG	GCC	GAC	ZF70	ZF58	ZF99	ZF58	ZF73	ZF65
8693L+R	5'-CCT TCC CCG TTG CCA GTC TCGATC **CGC CCC GTC GTT CCT GGC**-3'	GGC	CCT	GTT	GTC	CCC	CGC	ZF61	ZF96	ZF68	ZF69	ZF94	ZF98
	3'-**GGA AGG GGC AAC GGT CAG** AGCTAG GCG GGG CAG CAA GGA CCG-5'	AGG	GGA	CGG	CAA	TGG	GAC	ZF84	ZF59	ZF99	ZF89	ZF106	ZF65
8996L+R	5'-TCC CCG TTG CCA GTC TCG ATCCGC **CCC GTC GTT CCT GGC CCT**-3'	CCT	GGC	CCT	GTT	GTC	CCC	ZF96	ZF61	ZF96	ZF68	ZF69	ZF94
	3'-**AGG GGC AAC GGT CAG AGC** TAGGCG GGG CAG CAA GGA CCG GGA-5'	GGA	CGG	CAA	TGG	GAC	CGA	ZF59	ZF99	ZF89	ZF106	ZF65	ZF97
9299L+R	5'-CCG TTG CCA GTC TCG ATC CGCCCC **GTC GTT CCT GGC CCT GGG**-3'	GGG	CCT	GGC	CCT	GTT	GTC	ZF58	ZF96	ZF61	ZF96	ZF68	ZF69
	3'-**GGC AAC GGT CAG AGC TAG** GCGGGG CAG CAA GGA CCG GGA CCC-5'	CGG	CAA	TGG	GAC	CGA	GAT	ZF99	ZF89	ZF106	ZF65	ZF97	ZF64
95102L+R	5'-TTG CCA GTC TCG ATC CGC CCCGTC **GTT CCT GGC CCT GGG CTT**-3'	CTT	GGG	CCT	GGC	CCT	GTT	ZF103	ZF58	ZF96	ZF61	ZF96	ZF68
	3'-**AAC GGT CAG AGC TAG GCG** GGGCAG CAA GGA CCG GGA CCC GAA-5'	CAA	TGG	GAC	CGA	GAT	GCG	ZF89	ZF106	ZF65	ZF97	ZF64	ZF70
126133L+R	5'-GCC CTG GGC TTT GCC ACC CTATGC **TGA CAC CCC GTC CCA GTC**-3'	GTC	CCA	GTC	CCC	CAC	TGA	ZF69	ZF93	ZF69	ZF94	ZF90	ZF105
	3'-**CGG GAC CCG AAA CGG TGG** GATACG ACT GTG GGG CAG GGT CAG-5'	GGC	CAG	GCC	AAA	GGC	GGT	ZF61	ZF91	ZF73	ZF74	ZF61	ZF60
129136L+R	5'-CTG GGC TTT GCC ACC CTA TGCTGA **CAC CCC GTC CCA GTC CCC**-3'	CCC	GTC	CCA	GTC	CCC	CAC	ZF94	ZF69	ZF93	ZF69	ZF94	ZF90
	3'-**GAC CCG AAA CGG TGG GAT** ACGACT GTG GGG CAG GGT CAG GGG-5'	CAG	GCC	AAA	GGC	GGT	TAG	ZF91	ZF73	ZF74	ZF61	ZF60	ZF104
132139L+R	5'-GGC TTT GCC ACC CTA TGC TGACAC **CCC GTC CCA GTC CCC CTT**-3'	CTT	CCC	GTC	CCA	GTC	CCC	ZF103	ZF94	ZF69	ZF93	ZF69	ZF94
	3'-**CCG AAA CGG TGG GAT ACG** ACTGTG GGG CAG GGT CAG GGG GAA-5'	GCC	AAA	GGC	GGT	TAG	GCA	ZF73	ZF74	ZF61	ZF60	ZF104	ZF71
3138L+R	5'-TTC AGG TTT ACT CAC GTC ATCCAG **CAG AGA ATG GAA AGT CAA**-3'	CAA	AGT	GAA	ATG	AGA	CAG	ZF89	ZF85	ZF63	ZF87	ZF82	ZF91
	3'-**AAG TCC AAA TGA GTG CAG** TAGGTC GTC TCT TAC CTT TCA GTT-5'	GAA	CCT	AAA	AGT	GTG	GAC	ZF63	ZF96	ZF74	ZF85	ZF66	ZF65

Table S2. Primer sequences

Primer	Sequences (5' to 3')	Applications
Finger 1 Fw	GGCTACGGTCTCCCACCTAATACGACTCACTATAGGTCTAGACCCAAGCCTTACAAATGCCCAGAATG	Construction
Finger 1 Rv	CCGATGGGTCTCCTTCTCCGGTATGTGTTCTCTGGTG	Construction
Finger 2 Fw	GGCTACGGTCTCCAGAAAAGCCTTACAAATGCCCAGAATG	Construction
Finger 2 Rv	CCGATGGGTCTCCCTGTCCGGTATGTGTTCTCTGGTG	Construction
Finger 3 Fw	GGCTACGGTCTCCACAGAAGCCTTACAAATGCCCAGAATG	Construction
Finger 3 Rv	CCGATGGGTCTCCCTCACCGGTATGTGTTCTCTGGTG	Construction
Finger 4 Fw	GGCTACGGTCTCCTGAGAAGCCTTACAAATGCCCAGAATG	Construction
Finger 4 Rv	CCGATGGGTCTCCTTGGCCGGTATGTGTTCTCTGGTG	Construction
Finger 5 Fw	GGCTACGGTCTCCCCAAAAGCCTTACAAATGCCCAGAATG	Construction
Finger 5 Rv	CCGATGGGTCTCCCTCCCCGGTATGTGTTCTCTGGTG	Construction
Finger 6 Fw	GGCTACGGTCTCCGGAGAAGCCTTACAAATGCCCAGAATG	Construction
Finger 6 Rv	CCGATGGGTCTCCGTGGATTTAGGTGACACTATAGAGGATCCACCGGTATGTGTTCTCTGGTG	Construction
T2027 Fw	GTCGcagcgcccccgcccggcgtctcccggggccaggtccaccct	Construction
T2027 Rv	CGGTagggtggacctggccccgggagacgccgggcgggggcgctg	Construction
T9299 Fw	GTCGccgttgccagtctcgatccgccccgtcgttcctggccctggg	Construction
T9299 Rv	CGGTcccagggccaggaacgacggggcggatcgagactggcaacgg	Construction
T9299v2 Fw	GTCGcccgttgccagtctcgatccgccccgtcgttcctggccctggg	Construction
T9299v2 Rv	CGGTcccagggccaggaacgacggggcggatcgagactggcaacggg	Construction

Table S3. Primer sequences

Primer	Sequences (5' to 3')	Applications

AAVS-1 Fw	cgatgtccggagaggatggc	T7E1 assay
AAVS-1 Rv	gatggtaaggaggactgcatgg	T7E1 assay
B2M Fw	ggtgcctgatatagcttgac	T7E1 assay
B2M Rv	cctgacaatcccaatatgca	T7E1 assay
2027ot1 fw	GAAGAGAATGAAGCGGCGGC	Off-target analysis
2027ot1 rv	ACTGACACCGAGACAGAGCA	Off-target analysis
2027ot2 fw	GAGGAGGCTCCCATCTGGCA	Off-target analysis
2027ot2 rv	TGTCTGAGGCAGGGGTAACC	Off-target analysis
9299ot1 fw	GATGGCTCCACCAACCATCC	Off-target analysis
9299ot1 rv	CAATCACTGAGGCAGGGAGC	Off-target analysis
9299ot2 fw	GGGCTCCAAGCAGACTGAAGT	Off-target analysis
9299ot2 rv	CCCACCGGGAGAAGCTTATC	Off-target analysis
9299v2ot1 fw	CCGCGCAGGATCCATTTTG	Off-target analysis
9299v2ot1 rv	CATCTTCCAGCACCTGCCAC	Off-target analysis
9299v2ot2 fw	GGGCTCCAAGCAGACTGAAGT	Off-target analysis
9299v2ot2 rv	CCCACCGGGAGAAGCTTATC	Off-target analysis

Table S4. Amino acid comparisons of 2027R and Zif268 at position 1 or 5 of the amino acid residues in the ZFs

2027R	Zif268	Amino acid position (-1－6)
**Finger 1**		
K49	S19	1
T53	T23	5
**Finger 2**		
K77	S47	1
T81	T51	5
**Finger 3**		
P105	S75	1
V109	K79	5
**Finger 4**		
A133	—	1
T137	—	5
**Finger 5**		
P161	—	1
V165	—	5
**Finger 6**		
S189	—	1
T193	—	5
